# Supplementary figures and images for: Detection and benchmarking of somatic mutations in cancer genomes using RNA-seq data
Source: PeerJ. 2018 Jul 31;6:e5362. doi: 10.7717/peerj.5362 (PMC6074801; doi:10.7717/peerj.5362)

Supplementary Figure S1

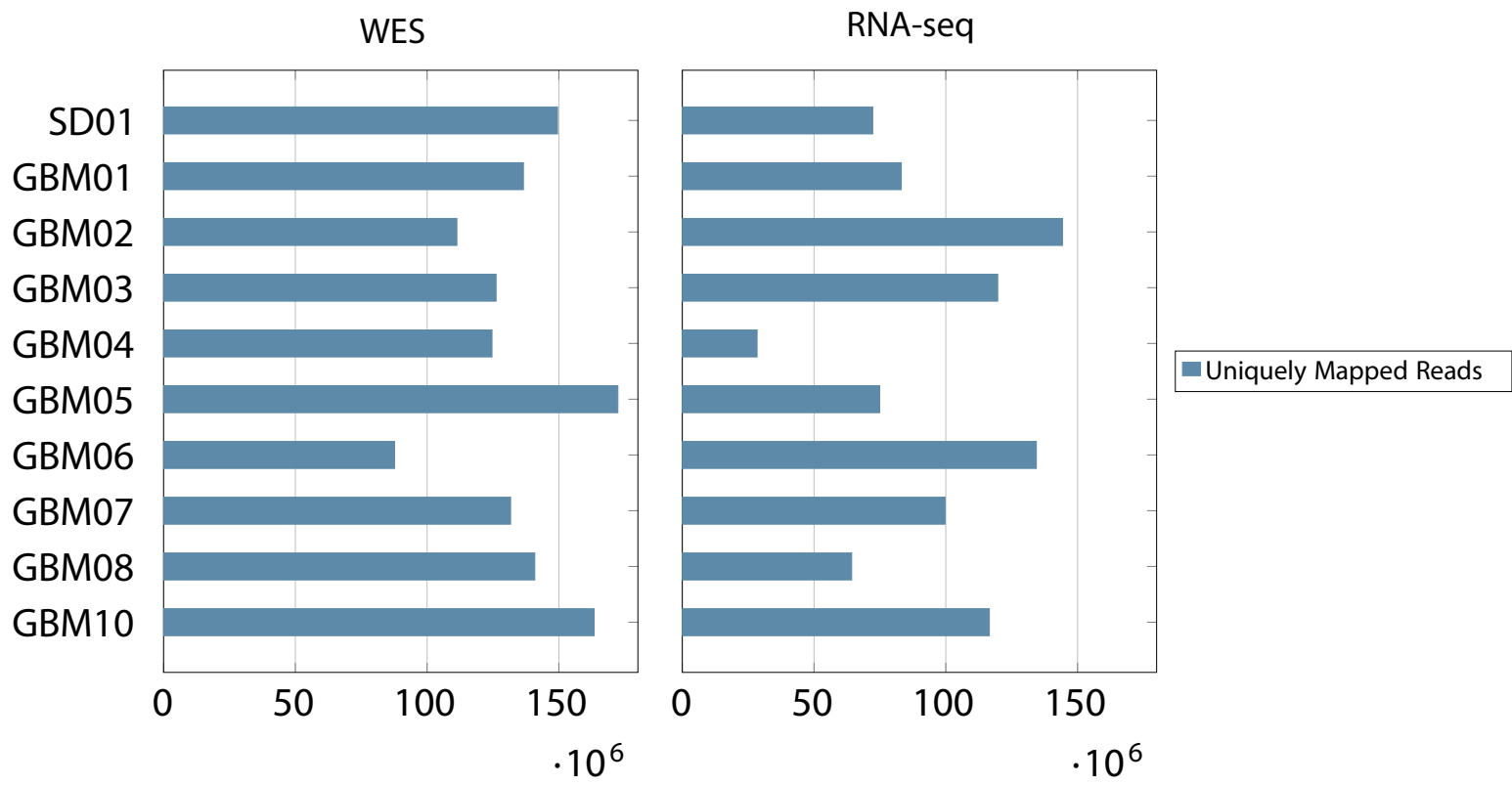

Supplement: Supplemental Information 1 — The number of reads accepted as uniquely mapped reads by STAR aligner is given for each sample, on the left for WES data and on the right for RNA-seq data. Counts are based on flag-stats given by samtools, done on BAM files before variant calling. [file peerj-06-5362-s001.pdf]

Supplementary Figure S2

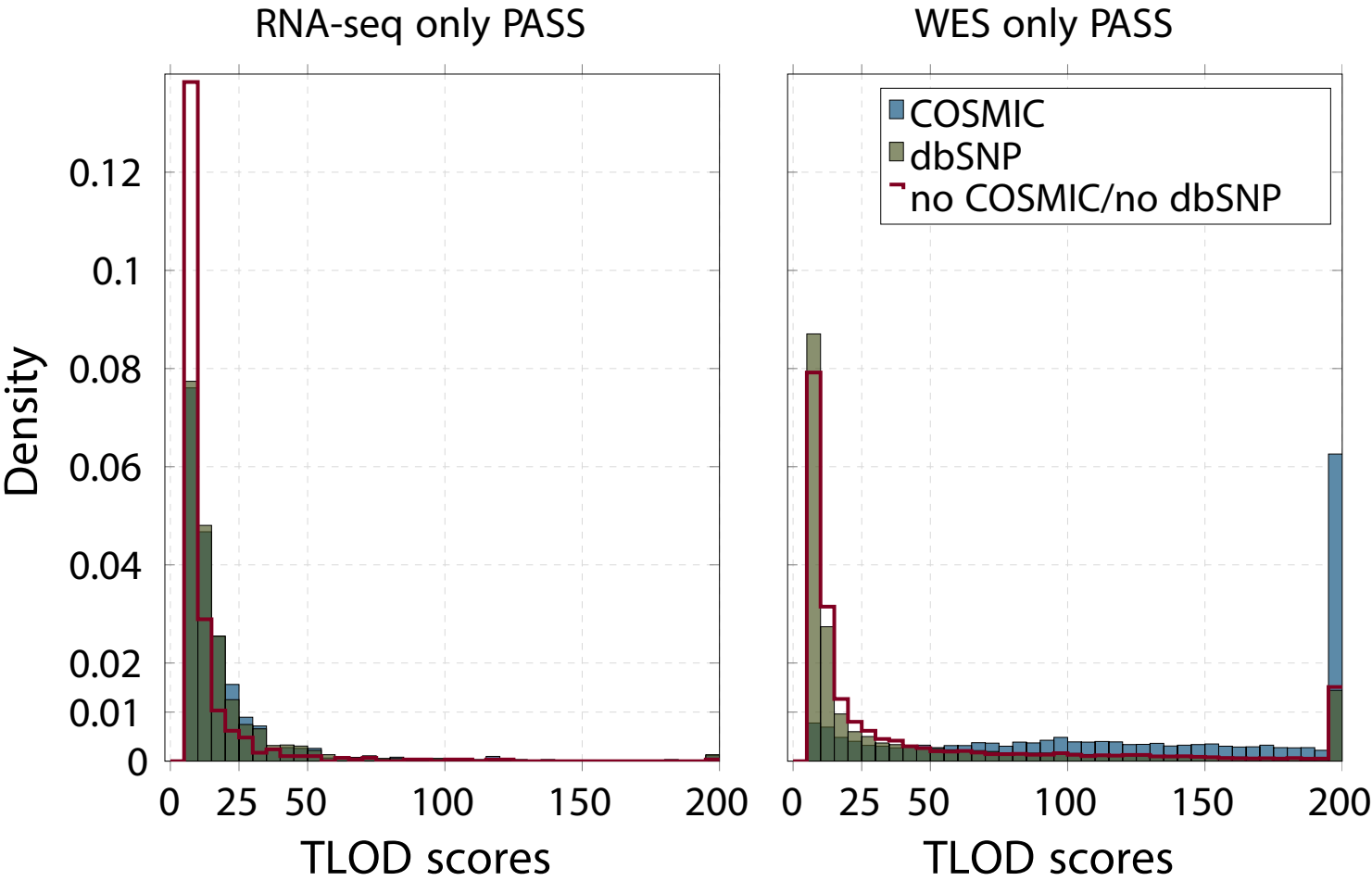

Supplement: Supplemental Information 2 — Histogram showing the distribution of TLOD scores of variants given by MuTect2 separated between different groups (COSMIC, dbSNP, not COSMIC/ not dbSNP). The Y-axis shows the proportion of mutations. [file peerj-06-5362-s002.pdf]

Supplementary Figure S3

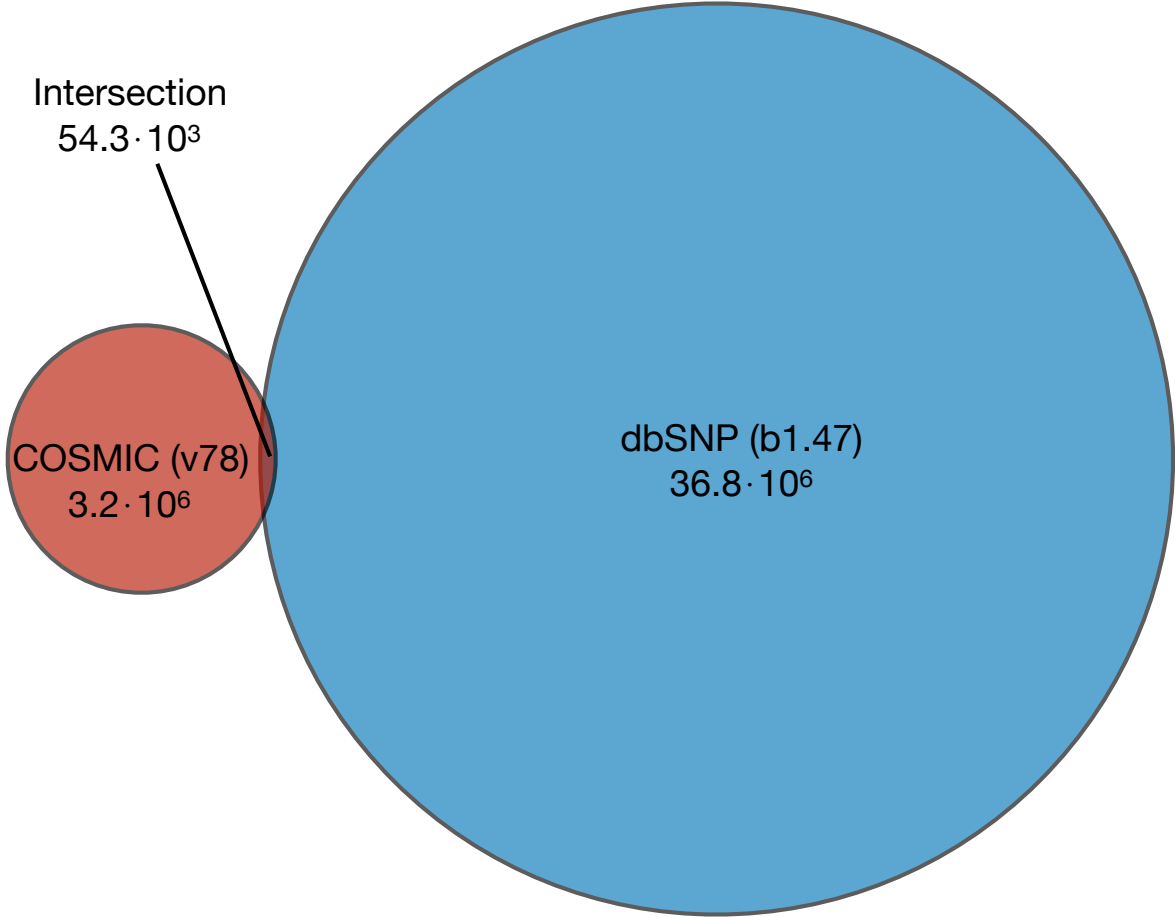

Supplement: Supplemental Information 3 — The intersection represents 0.15% of dbSNP (b147) variants and 1.65% of COSMIC (v78) variants. [file peerj-06-5362-s003.pdf]

Supplementary Figure S4

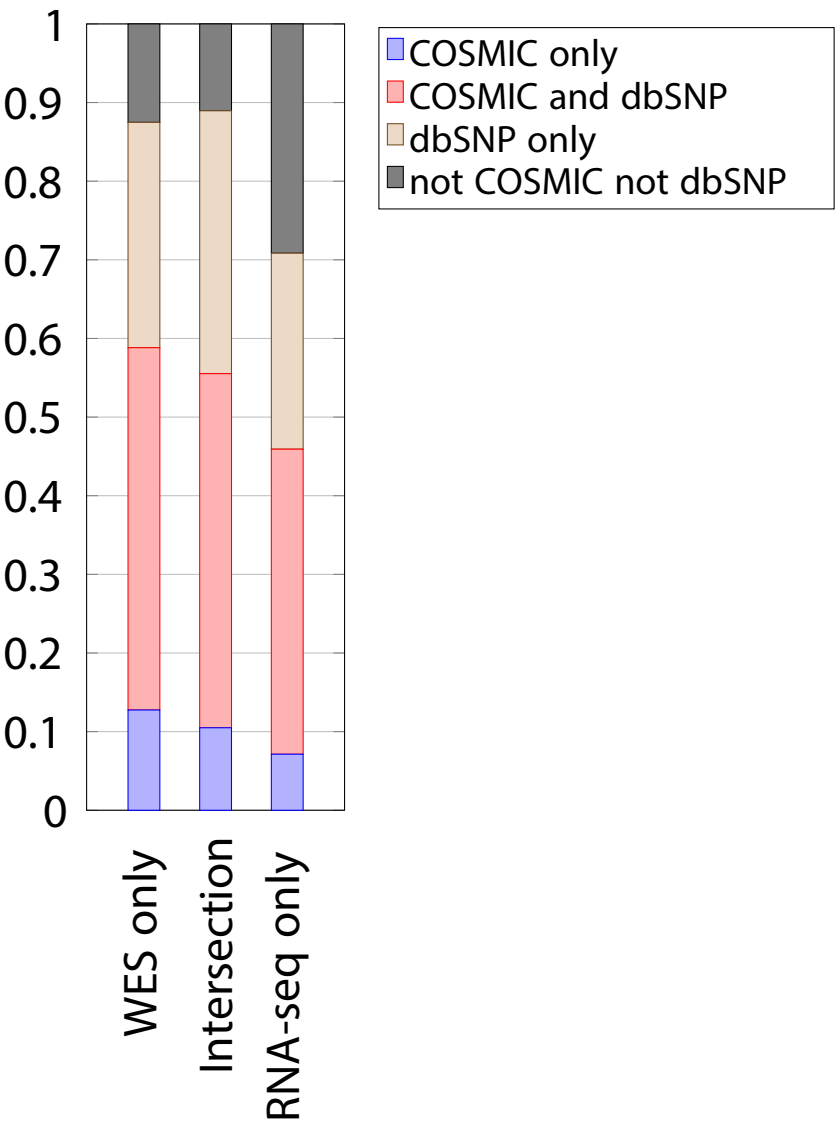

Supplement: Supplemental Information 4 — The Y-axis shows the proportion of variants indicated. [file peerj-06-5362-s004.pdf]

Supplementary Figure S5

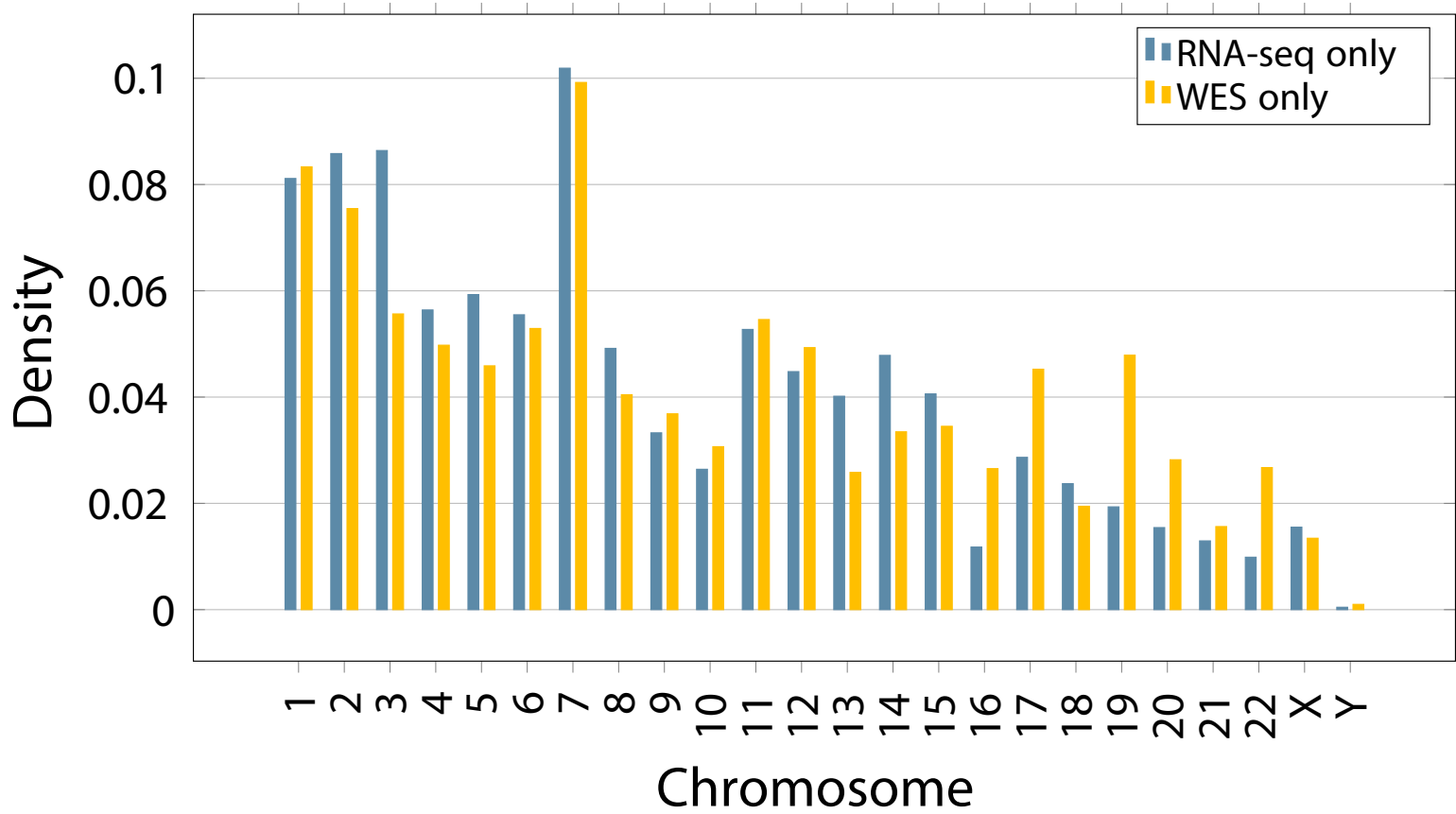

Supplement: Supplemental Information 5 — The Y-axis shows the proportion of mutations by chromosome. [file peerj-06-5362-s005.pdf]

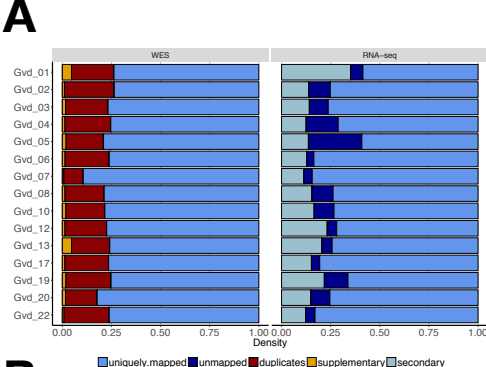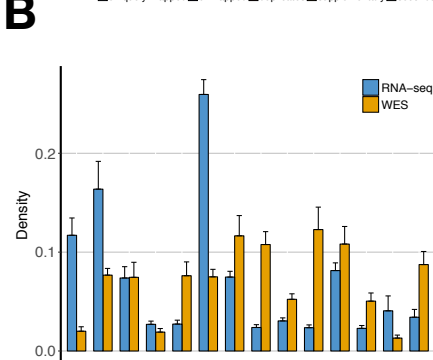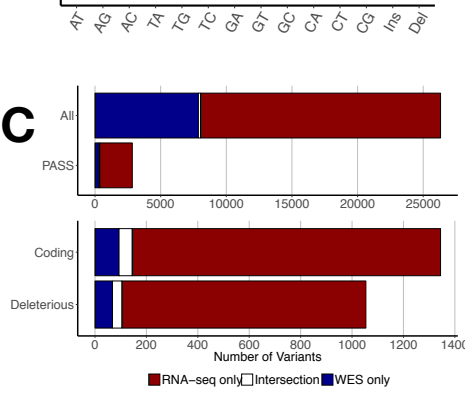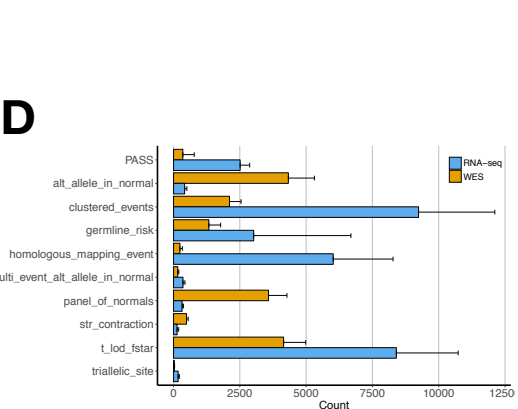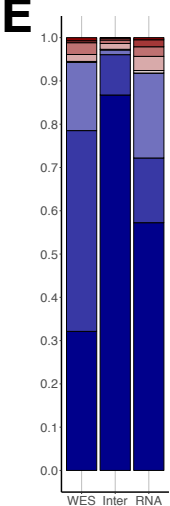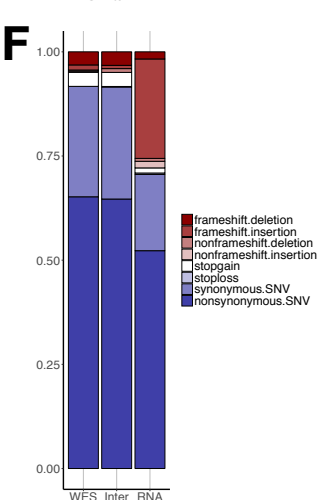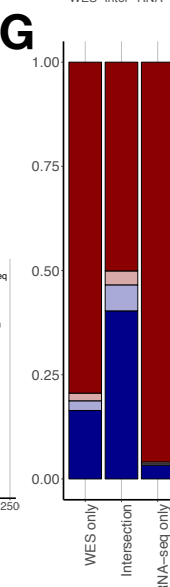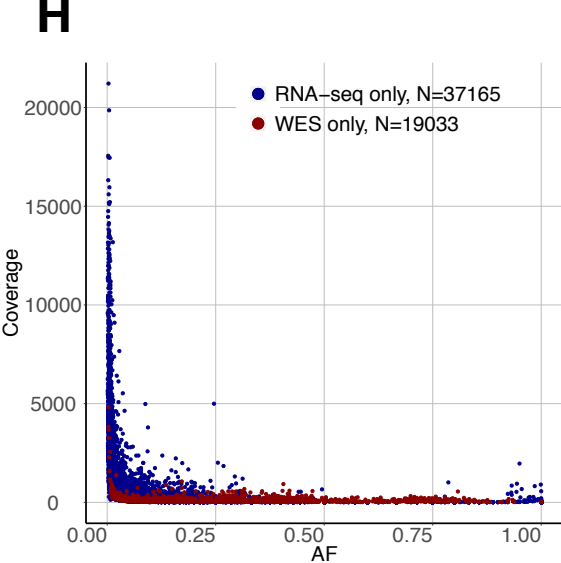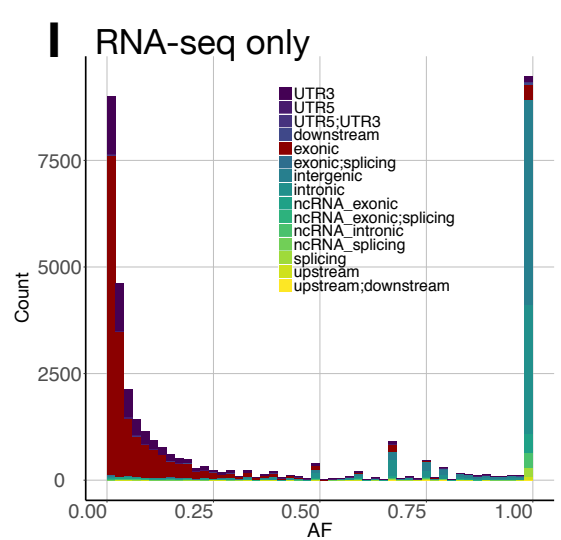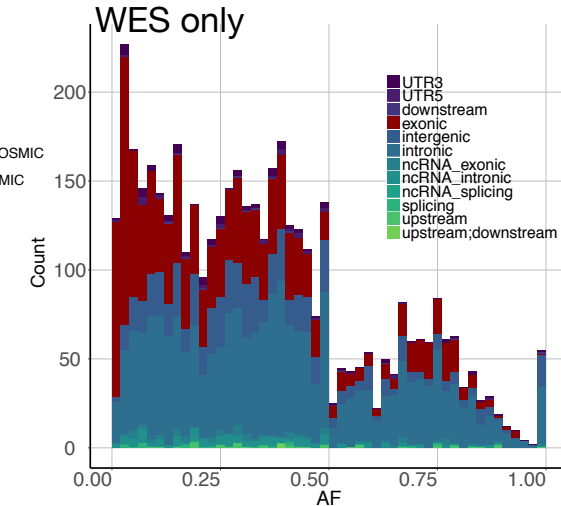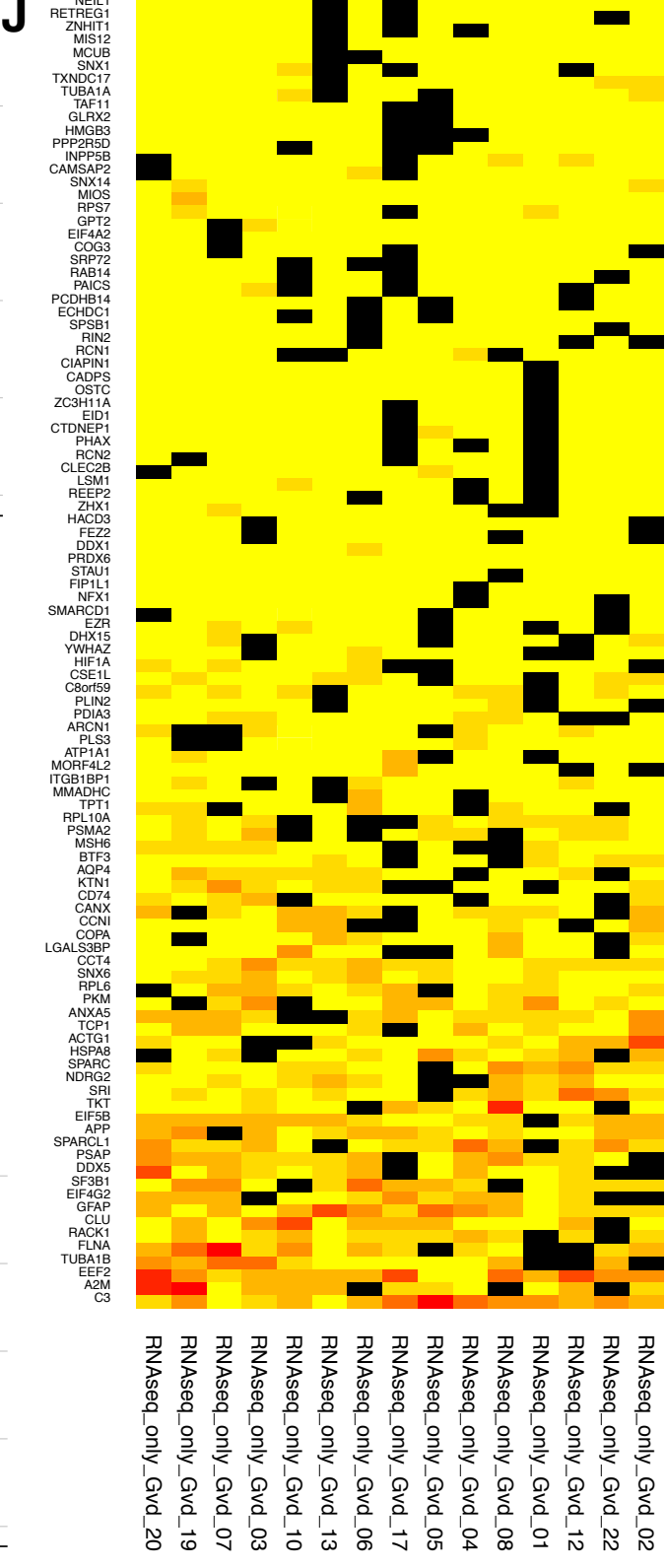

Supplement: Supplemental Information 6 — (A) Proportion of reads before variant calling. (B) Mutation spectrum indicating the type of base substitution in total RNA-seq and WES data. (C) Total number of variants for TCGA samples (averaged over 15 samples). Types of variants are as in Fig. 2D. (D) MuTect2 filtering statistics. Proportion of variants failing each MuTect2 filter, as described in Materials and Methods. (E) Genomic location of PASS variants, given as the average value over 15 samples. (F) Type of variants from coding regions, given as the average obtained over 15 samples. (G) Proportion of variants from coding regions included in COSMIC and/or dbSNP, given as the average obtained over 15 samples. (H) Scatter plot representing the fraction of the altered allele estimated from altered read fraction (allele fraction) versus coverage at the variant position (total number of reads). Merged data from 15 samples is shown. (I,J) Histograms of the distribution of allele fraction (AF) for the indicated classes of variants, from RNA-seq (I) and WES (J). Merged data from 15 samples is shown. (K) Heatmap of the most widely mutated genes across 15 samples in RNA-seq-only, similar to Fig. 4. Genes showing variants in at least 12 out of 15 samples are shown. [file peerj-06-5362-s006.pdf]
